# Clot embolization studies and computational framework for embolization in a canonical tube model

**DOI:** 10.1038/s41598-023-41825-8

**Published:** 2023-09-06

**Authors:** Nicolas Tobin, Menghan Li, Gretchen Hiller, Arash Azimi, Keefe B. Manning

**Affiliations:** 1 Department of Biomedical Engineering, The Pennsylvania State University, University Park, PA 16802 USA; 2 Department of Surgery, Penn State College of Medicine, Hershey, PA 17033 USA

**Keywords:** Experimental models of disease, Biomedical engineering, Computational science, Mechanical engineering

## Abstract

Despite recent advances in the development of computational methods of modeling thrombosis, relatively little effort has been made in developing methods of modeling blood clot embolization. Such a model would provide substantially greater understanding of the mechanics of embolization, as in-vitro and in-vivo characterization of embolization is difficult. Here, a method of computationally simulating embolization is developed. Experiments are performed of blood clots formed in a polycarbonate tube, where phosphate-buffered saline is run through the tube at increasing flow rates until the clot embolizes. The experiments revealed embolization can be initiated by leading edge and trailing edge detachment or by non-uniform detachment. Stress-relaxation experiments are also performed to establish values of constitutive parameters for subsequent simulations. The embolization in the tube is reproduced in silico using a multiphase volume-of-fluid approach, where the clot is modeled as viscoelastic. By varying the constitutive parameters at the wall, embolization can be reproduced in-silico at varying flow rates, and a range of constitutive parameters fitting the experiments is reported. Here, the leading edge embolization is simulated at flow rates consistent with the experiments demonstrating excellent agreement in this specific behavior.

## Introduction

The application of computational fluid dynamics (CFD) has made major advances in the realm of simulating thrombosis in recent years. For large blood vessels or artificial blood-contacting biomedical devices, research has focused on the development of single-scale methods of simulating thrombosis. Among the earliest attempts at CFD modeling of thrombosis was by Fogelson^1^, who developed a set of equations describing platelet activation by agonists, and platelet transport resulting in flow stasis via an applied force in the Navier–Stokes momentum equation. Goodman et al*.*^2^ applied modifications of Fogelson’s model by Sorensen et al*.*^3^ to thrombosis in flow through a set of cylinders with sudden contractions and found experimental and numerical agreement on the location of thrombus formation and the frequency of embolization. Fogelson and Guy^4^ developed an immersed-boundary method of microscale platelet aggregation modeling and showed that a coupled set of transport equations that described platelet-platelet link density and the cohesive stress holding a thrombus together had similar properties to the microscale model. Taylor et al.^5^ made modifications to the macroscale model developed by Fogelson and Guy^4^, altering the biochemistry and physical modeling of the clot, and applied their modifications to flow past a backward-facing step, matching numerical results with experimental data. Further modifications to the model of Taylor et al*.*^5^ were made by Yang et al*.*^6^ and Tobin and Manning^7^*.* Apart from modeling clot mechanics, CFD has even been shown useful in predicting regions of clot-formation in vivo^8,9^ and being applied to predict thrombosis is specific disease states^10^ and medical devices^11,12^. Thrombolysis has also seen significant efforts at computational modeling. Anand et al.^13^ developed a model for clot lysis, as well as efforts of modeling thrombolysis by Xu and collaborators^14,15^.

Despite the long history and relative success of computationally modeling clot formation and lysis, there have been fewer attempts at modeling embolization. Tobin and Manning^7^ investigated thrombosis and thromboembolism in turbulent flows. By incorporating the viscoelastic modeling of Fogelson and Guy^4^, they were able to produce instances of embolization in the backward-facing step used by Taylor et al*.*^5^ and Yang et al*.*^6^*.* This embolization behavior was not validated but could provide a framework. Good et al*.*^16^ also developed a computational modeling technique to simulate the use of an aspiration catheter in removing clots in acute ischemic stroke patients, modeling the adhesion of the clot to the vessel wall. Du et al.^17^ modeled embolization in a stenotic channel using a viscoelastic model and investigated the stability of thrombi at varying flow speeds.

A physics-based model informed by in vitro embolization data would allow for a much greater understanding of the mechanics of blood clot embolization, including adhesive forces anchoring a clot, the clot’s deformation as it breaks away, and the internal stretching the clot undergoes. In the current work, such an embolization model is detailed, and matched with in vitro embolization experiments, where clots are embolized by the flow of phosphate-buffered saline (PBS) in a plain tube. This computational model uses a volume-of-fluid approach, solving separate constitutive equations for the clot and the PBS on the same computational grid.

## Methods

### Bovine-blood experiments

Experiments were conducted on clots formed from freshly drawn bovine blood with approval from the Pennsylvania State University’s Institutional Animal Care and Use Committee and the appropriate procedures were in accordance with the relevant guidelines and regulations. Methods are reported in accordance with ARRIVE guidelines for the reporting of animal experiments.

### Blood collection and preparation

Blood was drawn from healthy adult female cattle (Bos taurus) into 450-mL bags containing 63 mL of citrate–phosphate-dextrose-adenine (CPDA-1) anticoagulant. The blood was centrifuged at 400*g* for 15 min, and the platelet-rich plasma (PRP) was separated from the red blood cells (RBCs). The RBCs were then centrifuged a second time at 1900*g* for 20 min, and the resultant platelet-poor plasma (PPP) was removed from the RBCs. The platelet concentration in the PRP was measured using a hemocytometer, and the PRP was diluted with PPP to achieve a plasma platelet concentration of 214,000 per microliter. Whole blood was then reconstituted to a hematocrit of 40% by mixing two parts RBCs to three parts reconstituted plasma. Immediately before forming clots, 0.5 M CaCl_2_ was mixed with the blood to achieve a calcium concentration of 19 mM to counteract the effect of the anticoagulant.

### Compressive testing of cylindrical clots

To find constitutive parameters for the embolization model, clots were formed in well plates with a diameter of 15 mm. Immediately after recalcification, clots were allowed to form at 37 °C for 1 h. The clots were then loaded into a mechanical tester (Instron—Norwood, MA, USA). The height and radius of the clots were measured, and they were subsequently loaded in compression at a rate of 10% per second for 1 s, and then a 10% compressive strain was maintained for 30 s, giving stress relaxation curves that are used to fit constitutive parameters in subsequent CFD simulations.

### Embolization experiments

After recalcification, 160 µL of blood was placed carefully in the center of a straight polycarbonate connector (Qosina-Ronkonkoma, NY, USA) with an inner diameter (D) of 12.7 mm. Blood was pipetted into the connector through the Luer lock port, the ends were covered to prevent the clot from drying out, and the blood was left to clot for 1 h at 37 °C. At the end of 1 h, the length of the clots was measured. Although flow conditions significantly impact clot composition, static, whole-blood clots were used to better control their composition and mechanical properties.

**Figure 1:**
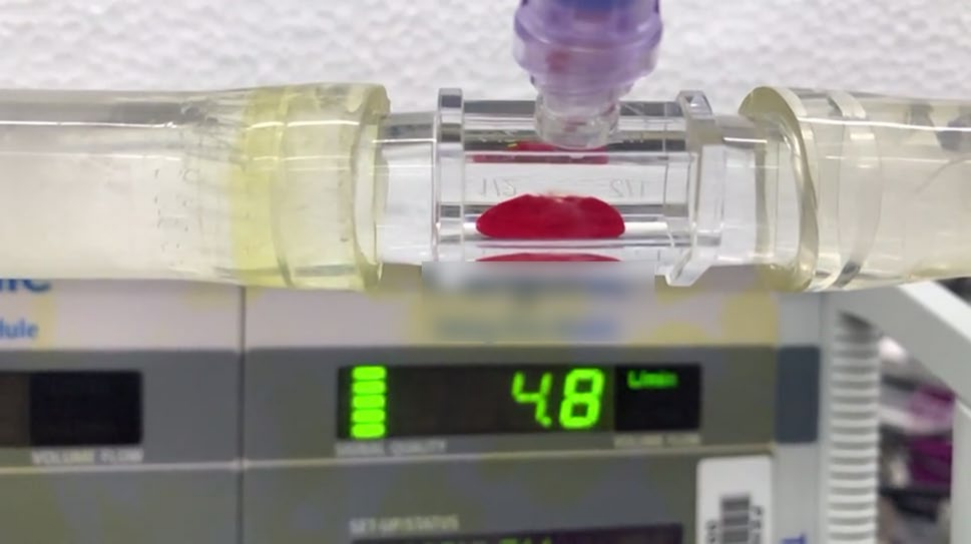
Blood clot formed in polycarbonate tube with flow probe screen in the background.

After clot formation, the polycarbonate connectors were installed in an open flow loop of phosphate-buffered saline (PBS). PBS was then run slowly through the loop to eliminate air from the system. The flow loop was run at a flow rate of approximately 0.5 L per minute (LPM), and the flow rate was increased by approximately 0.1 LPM every 5–10 s until embolization occurred. Flow rates were measured with an ultrasonic flow probe (Transonics, Inc.—Ithaca, NY, USA). Video was recorded of the embolization events using a mobile phone. The screen of the flow probe reader was within the frame of the video recordings to accurately record the flow rate at the point of embolization, which is the primary measured outcome. An example frame of the videos taken is shown in Fig. 1.

A total of 32 embolization events were recorded with blood from a total of seven animals. Samples from all animals were treated identically and there was no control group. The 32 events were accepted as they provided sufficient data to report statistics of embolization. Samples were excluded from the experiment in clots that failed to form, which was decided upon a priori. As there was only one experimental group, no randomization or blinding was performed.

### Micro-CT imaging

To further evaluate the shape of the formed blood clots, seven clots were scanned using Micro-CT (computed tomography) imaging at Penn State’s Huck Institute for Life Sciences. To prevent the clots from drying out, scanning was performed within 8 h of the clots forming. This was performed to observe the shapes of the clots to ensure that an idealized shape used in the simulation was not substantially different from the real clots. The micro-CT images of the clots were not used in the simulations.

### Computational fluid dynamics simulations

#### Computational grid

Concurrent simulations were run on two O–H or butterfly grid regions (topology depicted in Fig. 3) produced with OpenFOAM’s blockMesh utility, both with a diameter of 1.27 cm. The first grid region (region 1) was used to produce fully developed pipe flow which is used as an inlet boundary condition for the second region (region 2). Region 1 had grid stretching to ensure proper grid resolution near the wall. At the wall, the wall-normal grid spacing was 15 µm; the tangential grid spacing was approximately 332 µm, and the streamwise spacing was approximately 1411 µm. At the highest flow rate (10 LPM), this corresponded to a wall-normal Δy^+^ of approximately 0.9, a streamwise Δx^+^ of approximately 84, and a spanwise Δz^+^ of approximately 19. Here, quantities with a plus superscript indicate grid spacings in wall units*, e.g.*, $\Delta {y}^{+}=\Delta y*{u}_{\tau }/\nu$, where ${u}_{\tau }=\sqrt{{\tau }_{w}/\rho }$ is the friction velocity, ${\tau }_{w}$ is the wall shear stress, and $\rho$ is the fluid density. These values are consistent with best practices for wall-resolved LES of pipe flows^18^. Region 1 had a length of 12.7 cm (10D) and consisted of 469,800 finite-volume elements.

**Figure 2:**
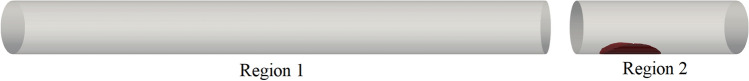
Computational regions 1 and 2. Region 1 provided a fully-developed pipe flow inlet boundary condition to region 2, which interpolated the velocity solution from the outlet of region 1 and every time point.

**Figure 3:**
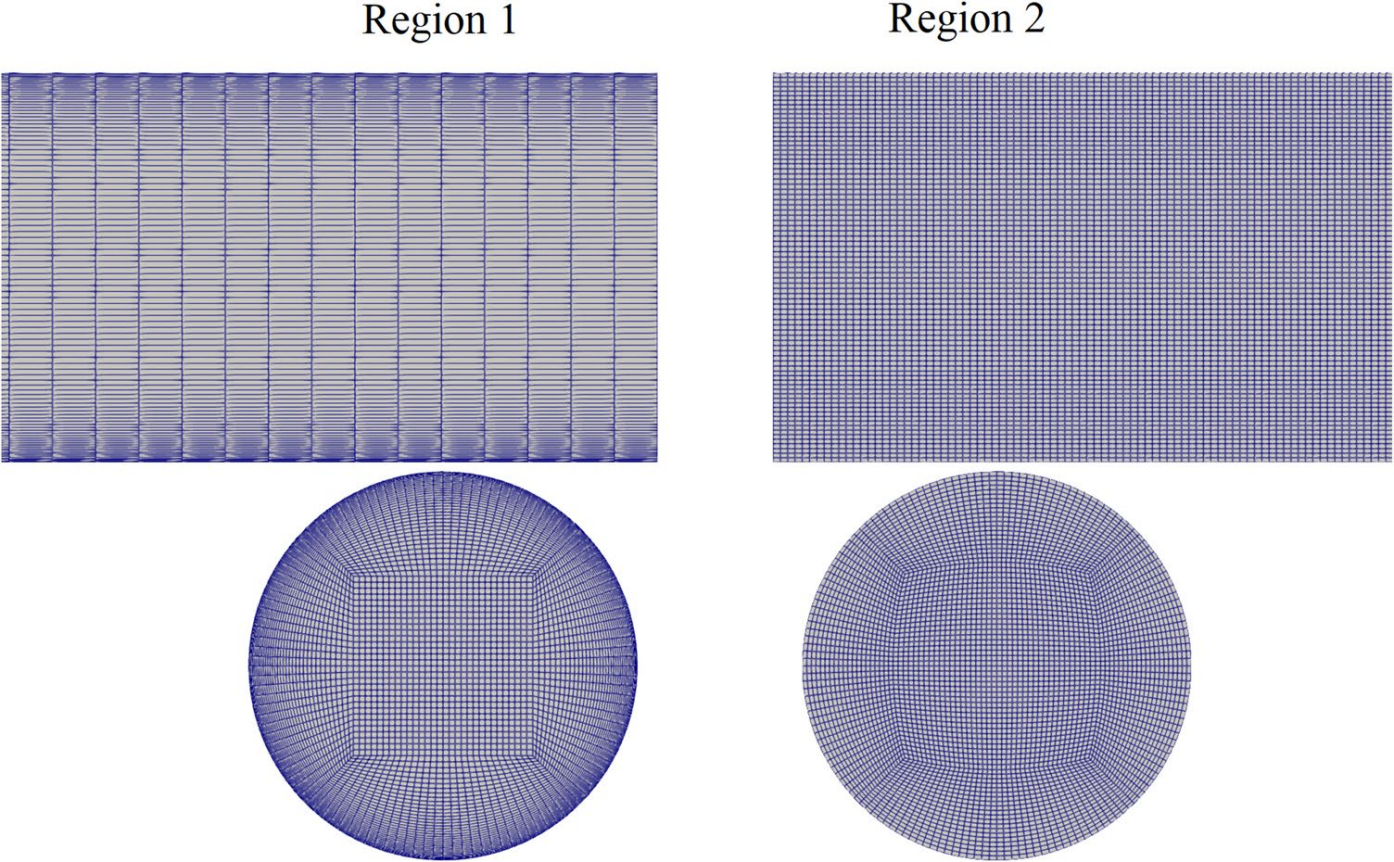
Computational meshes used in regions 1 and 2. Region 1 used grid stretching to achieve a small y + near the wall, while region 2 used more regularly shaped elements to improve solution stability for the viscoelastic equations.

Due to numerical instability observed in high-aspect-ratio grid volumes when solving the viscoelastic equations for clot transport, region 2 used uniform radial grid spacing in the outer O-grid portions (i.e., no grid stretching to achieve flatter elements at the wall) and a smaller streamwise grid spacing. The wall-normal grid spacing was approximately 70 µm, while the streamwise grid spacing was approximately 158 µm and the tangential grid spacing was approximately 166 µm. At the highest flow rate, this corresponded to a Δy^+^ of approximately 4.1, an Δx^+^ of approximately 9.4, and Δz^+^ of approximately 9.9. Region 2 had a length of 3.81 cm (3D) and consisted of 2,592,000 finite-volume elements. Regions 1 and 2 are depicted in Fig. 2. Their grids are depicted in Fig. 3.

#### Simulation details

Simulations are performed in OpenFOAM.

Detailed information on the implemented model and details specific to the simulation are provided in Supplementary Materials 3.

To capture the entire range of flow rates observed to produce embolization, five flow conditions were simulated. These conditions correspond to 1.9, 2.8, 4.2, 6.3, and 10 LPM. PBS was modeled with a kinematic viscosity of 1 × 10^–6^ m^2^/s, consistent with that of PBS at 20° Celsius. The flow rates corresponded to Reynolds numbers of 3175, 4674, 7023, 10,528, and 16,713, respectively, based on the kinematic viscosity of the PBS, the mean velocity in the tube, and the tube’s diameter. These flow rates also corresponded to wall shear rates of 1200, 1770, 2360, 3170, and 4440 s^−1^, respectively.

Region 1 used a periodic inlet velocity boundary condition with an enforced average velocity. That is, the velocity field prescribed at the inlet at each timestep was equal to the velocity field at the outlet allowing the velocity field to develop over long time scales and become fully developed. The velocity field at the inlet of region 2 was interpolated from the outlet of region 1. This allowed for a fully developed turbulent pipe flow to approach the clot while meeting the grid requirements of the two regions. The outlets of both regions 1 and 2 used constant pressure boundary conditions, setting the pressure equal to 0.

Precursor large-eddy simulations (LES) were run at each flow rate, using a wall-adapting local eddy viscosity (WALE) subgrid-scale model^19^. These precursor simulations were performed with the goal of preventing non-physical startup transients from impacting the clot and giving a physically realistic initial velocity field for embolization simulations. During these precursor simulations, no modeling was performed of the clot; the clot region had an enforced zero velocity, replicating a non-moving solid obstruction in the flow. The clot region was modeled as the intersection of the computational domain with a spanwise-oriented cylinder. The clot region had a height of 2.6 mm, a length of 13.8 mm, and a volume of 159 µL. To speed up the development of turbulent motions, the initial conditions in the velocity field for the precursor simulations had structured noise which was allowed to develop in time. To allow startup transients to pass and for turbulence to develop, simulations were performed for 100 characteristic timescales at each flow rate (T = D/U, where T is the characteristic timescale and U is the mean velocity at the inlet). Temporal averaging of the velocity and pressure fields was then performed for an additional 200 characteristic timescales. During precursor simulations, a Courant number of less than 0.5 was enforced throughout the domain. This process of running a precursor simulation provided a physically realistic initial condition for the embolization simulations and allowed for the characterization of the forces acting on the clot.

After the initial 300 characteristic timescales were simulated in the precursor simulation, the velocity field at the end of the simulation was used as an initial condition for multiphase volume-of-fluid (VOF) embolization simulations including a Newtonian phase for the PBS and a Phan-Thien-Tanner (PTT) viscoelastic constitutive model in the clot phase^20^. Using a VOF approach, these simulations solved a single set of momentum and continuity equations on a single structured grid, along with a transport equation for a volume fraction $\alpha$. OpenFOAM’s interface compression scheme was used to maintain a sharp interface between the PBS and clot. This volume fraction was used to designate whether a grid element was clot or PBS. The local stress term switched between the Newtonian constitutive model and the PTT constitutive model depending on the volume fraction. The initial condition of the volume fraction was set so that the same zero-flow region from the precursor simulations was designated as a clot, with $\alpha =1$. Because OpenFOAM does not have the capability to run multiphase simulations where one phase is Newtonian and the other is viscoelastic, simulations relied on the third-party library of solvers rheoTool^21^. During the embolization simulations, there was no subgrid-scale model applied to avoid complications arising with the interaction between the subgrid-scale model and the viscoelastic model. The polymeric stress tensor for the PTT model followed an evolution equation as in Eq. (1). During embolization simulations, an interface Courant number of 0.01 was enforced.

1 $$\lambda \left[\frac{\partial {\tau }_{p}}{\partial t}+u\cdot \frac{\partial {\tau }_{p}}{\partial x}-{\tau }_{p}\nabla u-{\left(\nabla u\right)}^{T}{\tau }_{p}+\zeta ({\tau }_{p}E+E{\tau }_{p})\right]={\eta }_{p}\left(\nabla u+{\left(\nabla u\right)}^{T}\right)-f\left({\tau }_{p}\right){\tau }_{p}$$

In Eq. (1), $\lambda$ is the relaxation timescale, ${\tau }_{p}$ is the polymeric stress tensor, ${\eta }_{p}$ is the polymeric viscosity, $u$ is the velocity vector, and $\nabla u$ is the gradient of the velocity. The left-hand side of Eq. (1) constitutes a Gordon-Schowalter derivative, where $E$ is the symmetric part of the velocity gradient tensor and $\zeta$ is a parameter controlling non-affine deformation. In this work, $\zeta$ is set to zero, indicating an assumption of affine deformation of the polymer network. This assumption was also made by Storm et al*.*^22^, who discuss its implications. The polymeric viscosity and relaxation timescale were fit to the experimental compression testing data. The function $f({\tau }_{p})$ is a destruction function as given in Eq. (2), first proposed by Phan-Thien^23^ and tends to reduce the magnitude of the polymeric stress when large stresses accumulate, replicating the breakage of a subgrid fiber network (a fibrin mesh or adhered platelets in the case of a blood clot).

In Eq. (2), $\varepsilon$ is an extensibility parameter which is typically set to 0.01, as originally suggested by Phan-Thien and Tanner^20^ and is in the range of values commonly used by other authors^24^. This value was used for the current simulations and ensured that clots were more prone to breaking at the wall than in their volumes. The quantity ${\eta }_{p}/\lambda$ has units of stress and may be understood as an elastic modulus^25^ for small strains and small times. Therefore, the quantity $\frac{\lambda }{{\eta }_{p}}tr({\tau }_{p})$, which is found in the argument of the breakage function, is a measure of strain. Conceptually, this causes the subgrid fiber network to break when the viscoelastic material is subjected to large strain. To model the effect of clots breaking away from the wall, wall-adjacent grid volumes used a different value of the extensibility parameter, ${\varepsilon }_{w}$. The introduction of a higher extensibility parameter at the wall is motivated by the observation that clots primarily break away from the wall experimentally rather than break apart. This varying extensibility parameter may conceptually correspond to the types of bonds holding a clot to a wall as opposed to holding a clot together, for instance platelets and fibrin. Similar attempts at modeling wall adhesion separately from clot cohesion are detailed in Fogelson and Guy^4^ and Good et al.^16^ At each flow rate, different values of ${\varepsilon }_{w}$ were simulated to find bounds for the threshold wall extensibility parameter where embolization occurs.

2 $$f\left({\tau }_{p}\right)=\mathrm{exp}\left(\frac{\varepsilon \lambda }{{\eta }_{p}}tr({\tau }_{p})\right)$$

#### Compression test simulation

To replicate the compression testing and find values for ${\eta }_{p}$ and $\lambda$, a radially symmetric multiphase simulation was performed. This conceptually replicates the compression testing, with a cylindrical clot compressed by an upper moving wall. The clot was treated as a PTT viscoelastic material, and the surrounding fluid was modeled as air with a density of 1.204 kg/m^3^ and a dynamic viscosity of 1.825 Pa-s. The mesh consisted of a 2-degree slice of a clot 15 mm in diameter and 10 mm in height, with OpenFOAM’s built-in wedge boundary condition on the front and back faces. The mesh used 3600 finite volume cells with a concentrated mesh resolution at the border between clot and air, and near the top and bottom of the clot. Compression was achieved with a dynamic mesh, where the top wall displaced downward at every timestep, compressing the clot at a constant rate of displacement at the top. The dynamic mesh motion was enabled for the first 1 s until a strain of 10% was achieved (corresponding to a compressed clot height of 9 mm), and the dynamic mesh motion was then turned off for an additional 30 s. The resultant velocity gradient within the clot produced a stress within the clot as per Eq. (1), and the total force exerted by the clot onto the bottom wall was recorded by integration of the pressure plus the normal component of the traction vector at every timepoint during simulation runtime.

#### Grid dependence study

To assess the impact of grid resolution on embolization and other simulation outputs, the 1.9 LPM case was simulated with a grid spacing in region 2 that was decreased by a factor of 1.5, resulting in 8,748,000 finite-volume elements. Embolization was simulated using wall extensibility parameters determined by the lower-resolution grid to ensure that the true threshold value stayed within the same bounds. Due to the high computational cost, grid resolution characterization was not repeated at higher flow rates.

## Results

### In vitro tube embolization experiments

Across all 32 samples, the mean flow rate at embolization was 5.1 LPM, and the standard deviation of flow rate at embolization was 1.1 LPM. Embolization occurred at flow rates ranging from 2.3 to 7.5 LPM. The average length of clots after formation was 14.2 mm with a standard deviation of 1.7 mm.

Video recordings indicate a wide range of embolization behaviors. Embolization was typically initiated by detachment of the trailing edge of the clot, i.e., on the very end of the downstream edge of the clot. Of the 32 recorded embolization events, 23 were initiated by detachment of the trailing edge, one was initiated by detachment of the leading edge, and in eight embolizations, it was unclear whether the leading or trailing edge detached first. During embolization initiated by detachment of the trailing edge, there was variability in embolization behavior. In most instances, the trailing edge detached, and the entire clot subsequently detached shortly after. However, in a small number of embolization events, the clot remained attached at the leading edge and underwent a “flag-waving” motion, where the trailing edge underwent large repeated deformations until the leading-edge attachment point failed. An example of the “flag-waving” embolization is shown in Supplementary Materials 1.

**Figure 4:**
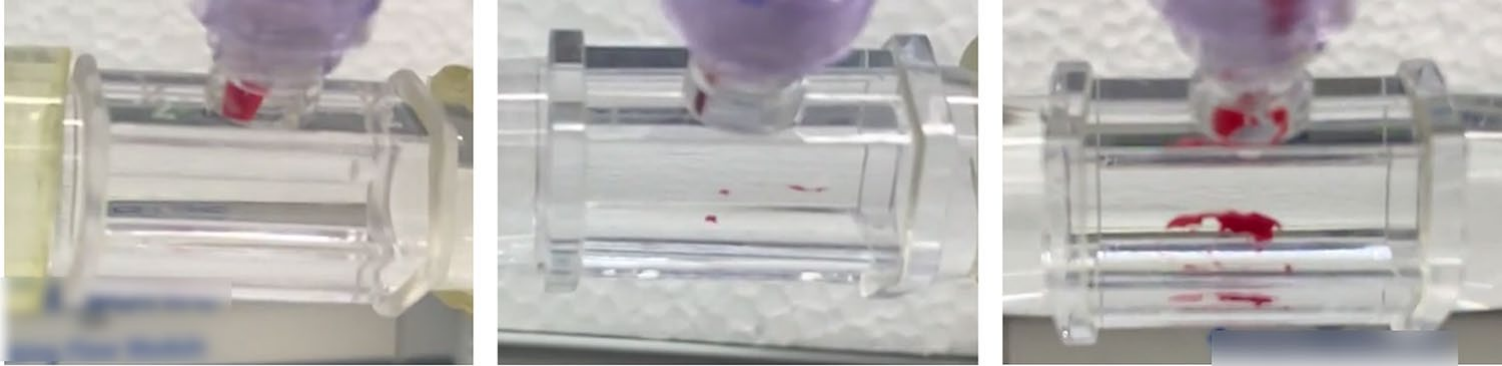
Varying degrees of clot remnants. Left—an embolization event that has left the tube nearly clear of clotted material. Some blood remains in the Luer lock through which the blood was deposited when forming the clots. Center—several small remnants are left. Right—one large remnant is left after the majority of the clot embolized.

The completeness of embolization also varied. In 16 embolization events, the entire clot embolized in a single piece and left no remnants on the polycarbonate surface. In eight embolization events, small sub-millimeter remnants were left after the majority of the clot embolized in a single event. In four embolization events, large remnants similar in length to the length of the clot were left after the clot embolized. In another four embolization events, the clot disintegrated into multiple pieces which embolized separately. Examples of varying degrees of clot remnants are depicted in Fig. 4.

### Clot compression experiments and simulations

**Figure 5:**
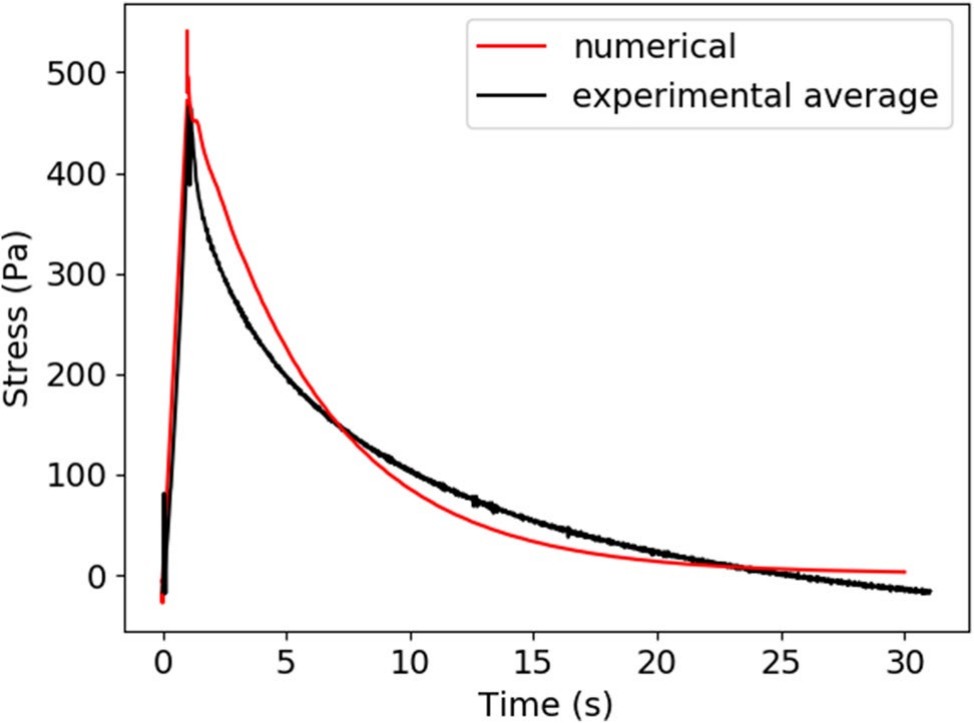
Comparison between experimental and numerical stress-relaxation curves used to fit constitutive parameters in PTT model.

Experimental compression data showed a typical stress-relaxation curve, achieving a local maximum stress value at 1 s when the compression ended. The mean stress-relaxation curve is reported in Fig. 5 along with the simulated stress-relaxation curve using constitutive parameters ${\eta }_{p}=2.1\times {10}^{4}$ Pa-s and $\lambda =6$ s. This corresponds to an elastic modulus of 3.5 kPa, which is similar to other values for fresh formed clots in the literature. Dempfle et al*.*^26^ found elastic moduli ranging from approximately 700 Pa to 3.2 kPa, while Mfoumou et al*.*^27^ found elastic moduli ranging from 1.0 to 25.0 kPa. Good^28^ fitted a Prony series to cyclically loaded clots, which includes three time-relaxation constants. He found that the value of the time constant ranged from 0.16 to 18.1 s, in the range of the 6-s relaxation time found in the current work. These constitutive parameters give an R^2^ value of 0.94 between experimental and numerical stress time curves. Because these values provided moderate agreement in the stress-relaxation curve, they were used in subsequent embolization simulations as the embolization experiments used an identical protocol to the compression experiments.

### Micro-CT images of clots

**Figure 6:**
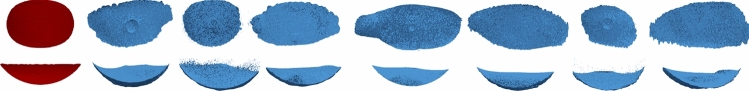
Computational and in vitro clots. Computational clot is colored in red, while in vitro clots are colored blue. The top row shows a top-down view of the clots. Below each top-down view is a front-on view of the same clot, showing its cross section in the direction of flow.

Figure 6 depicts the micro-CT images of seven in vitro clots along with the computational shape. The seven in vitro clots show significant variability in the shape of their footprints, though their lengths are all on the same order as the computational clot. The front-facing area is also well represented in the computational clot. The in vitro clots had an average height of 2.25 mm, ranging from 1.75 to 3.30 mm. The in silico clot had a height of 2.6 mm.

### Computational fluid dynamics simulations

#### Precursor simulations

Results from the precursor turbulent simulations are presented. Typical turbulent-flow notation is used, where overbars indicate a time-averaged quantity, primes indicate fluctuations from the time-averaged quantity, and plus superscripts indicate typical normalization by wall quantities for turbulent boundary layers.

**Figure 7:**
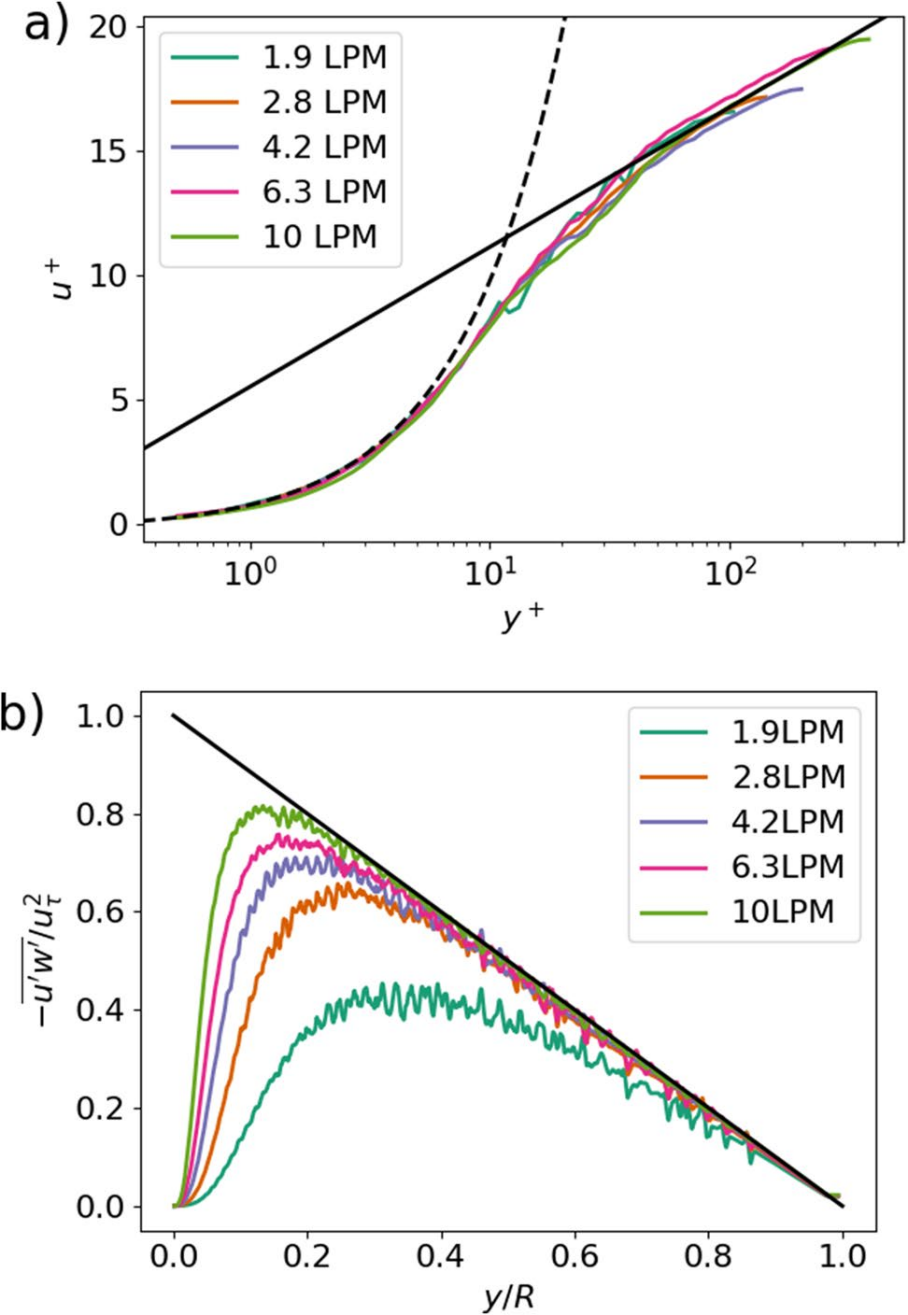
Characteristics of the turbulent pipe flow in region 1; (a) mean velocity normalized by friction velocity vs. distance from the wall in wall units. Solid black line indicates typical log-law profile, while dashed black line indicates typical linear viscous-sublayer profile; (b) streamwise-wall-normal component of Reynolds stress normalized by the square of the friction velocity. Black line indicates linearly decreasing Reynolds stress, typical of pipe flow in the log layer.

In region 1 during the precursor simulations, a fully developed turbulent pipe flow profile was observed at all flow rates. Normalized profiles of mean velocity and the resolved portion of the streamwise-wall-normal component of the Reynolds shear stress are depicted in Fig. 7. In this figure, ${u}^{+}=\overline{u }/{u}_{\tau }$. The normalized velocity is plotted as a function of distance from the wall in wall units ${y}^{+}=y*{u}_{\tau }/\nu$. In Fig. 2a, the dashed black line shows the line ${u}^{+}={y}^{+}$, which holds in the viscous sublayer of a fully developed turbulent boundary layer, and the solid black line shows the relation given in Eq. (3), the classical log-law of the wall. In Fig. 2b, the streamwise-wall-normal component of the Reynolds stress tensor is normalized by the square of the friction velocity and is plotted as a function of distance from the wall normalized by the radius of the tube. In Fig. 2b, the solid black line shows a linear decrease in resolved Reynolds stress toward zero at the center of the tube, a classical functional dependence of stress in pipe flows.

3 $${u}^{+}=\frac{1}{0.41}\mathrm{ln}\left({y}^{+}\right)+5.0$$

The total drag ${F}_{D}$ on the clot in the precursor simulations was calculated as given in Eq. (4), where $\overline{p }$ is the time-averaged pressure, ${\overrightarrow{n}}_{x}$ is the projection of the outward-facing normal of the clot’s surface $S$ in the direction of mean flow, and $\tilde{\sigma }$ is the time-averaged viscous stress tensor. The total drag force is reported as a function of flow rate in Table 1. In addition, Table 1 reports the coefficient of drag defined as in Eq. (5). In Eq. (5), $\rho$ = 1000 kg/m^3^ is the density of the PBS, and A = 0.181 cm^2^ is the clot’s area projected in the streamwise direction.

**Table 1:** Calculated drag exerted on clot during simulations with the corresponding drag coefficient.

	1.9 LPM	2.8 LPM	4.2 LPM	6.3 LPM	10 LPM
Drag Force (N)	$2.72\times {10}^{-4}$	$6.41\times {10}^{-4}$	$1.52\times {10}^{-3}$	$3.38\times {10}^{-3}$	$8.75\times {10}^{-3}$
Drag Coefficient (-)	0.480	0.522	0.547	0.543	0.557

4 $${F}_{D}={\int }_{S}(\overline{p}{\overrightarrow{n} }_{x}+\tilde{\sigma }\cdot {\overrightarrow{n}}_{x})dA$$

5 $${C}_{D}=\frac{2{F}_{D}}{\rho A{U}^{2}}$$

#### Embolization simulations

**Figure 8:**
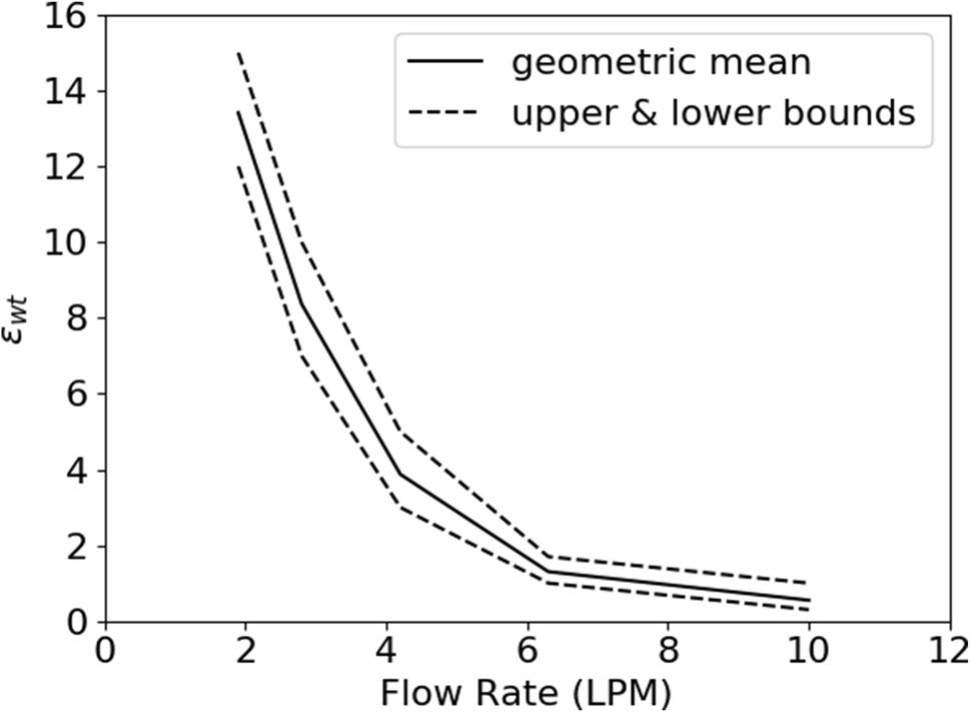
Threshold wall extensibility parameter as a function of flow rate. Dashed lines indicate upper and lower bounds of the threshold value, and the black line indicates the geometric mean of the upper and lower bounds.

Runtime processing was performed during all simulations to track the location of the leading edge of the clot. Tracking the leading edge of the clot indicated that simulations that did not lead to embolization maintained a leading-edge displacement of less than 5% of the clot’s length over five characteristic timescales. Therefore, a simulation is considered to fail to produce embolization if it maintains a leading-edge displacement of less than 5% of the clot’s length for at least ten characteristic timescales; otherwise the simulation is considered to have produced embolization. This allowed for more computationally efficient parameter sweeping, as modeling embolization becomes expensive as the clot detaches due to the need to maintain a low interface Courant number. This allows the threshold wall extensibility parameter ${\varepsilon }_{wt}$ to be bounded between the lowest value of ${\epsilon }_{w}$ that produces embolization and the highest values of ${\varepsilon }_{w}$ that does not produce embolization. Upper and lower bounds are depicted in Fig. 8 as dashed lines; the geometric mean of the upper and lower bounds is depicted in Fig. 8 as a solid black line.

A full example of clot embolization is included in Supplemental Materials 2, where embolization is depicted at a flow rate of 4.2 LPM and a wall extensibility parameter of 5.

#### Dynamics of embolization

**Figure 9:**
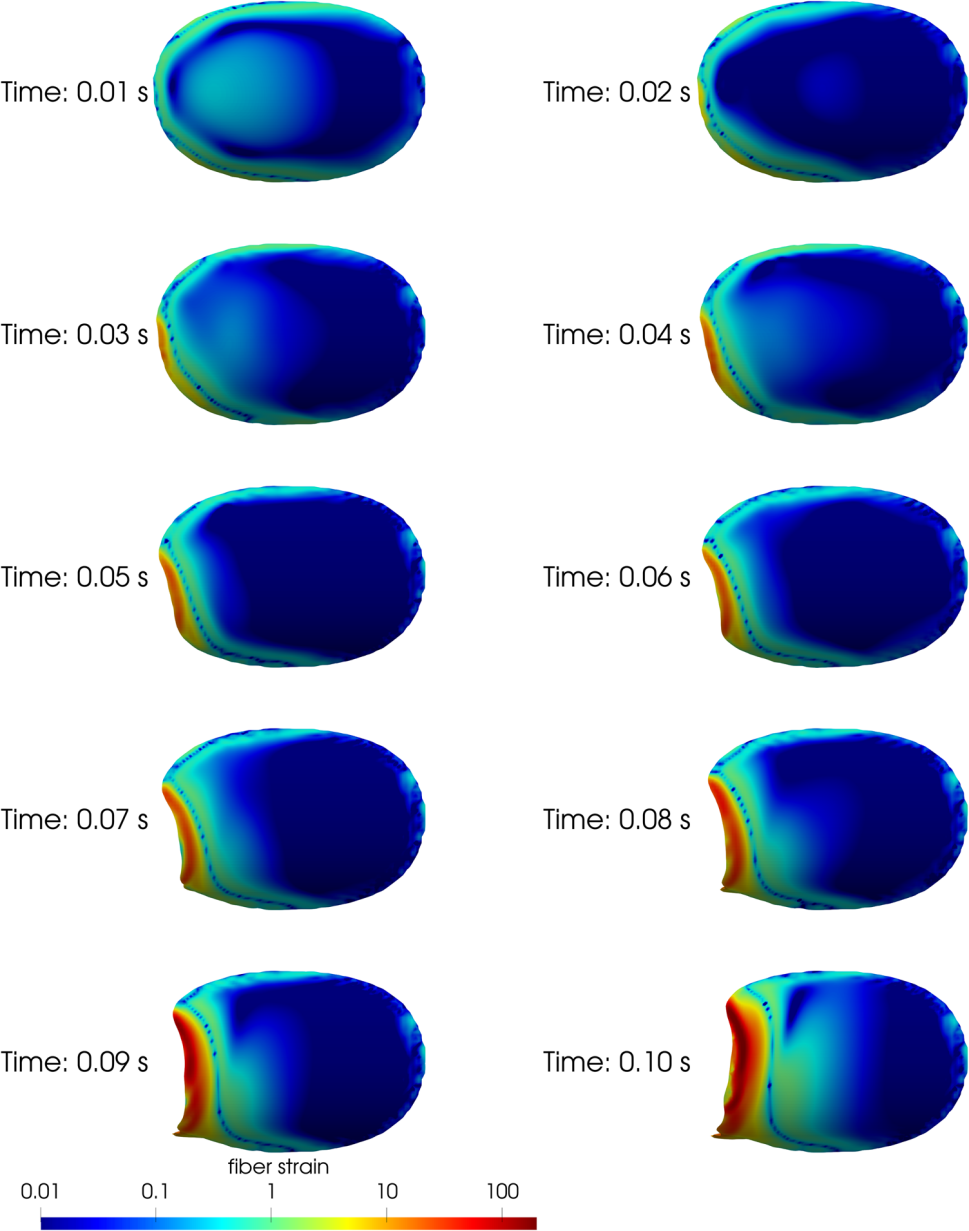
Early fiber strain concentrations at a flow rate of 4.2 LPM and ${\varepsilon }_{w}=5$ during an embolization event show that fiber strain is concentrated at the leading edge of the clot.

**Figure 10:**
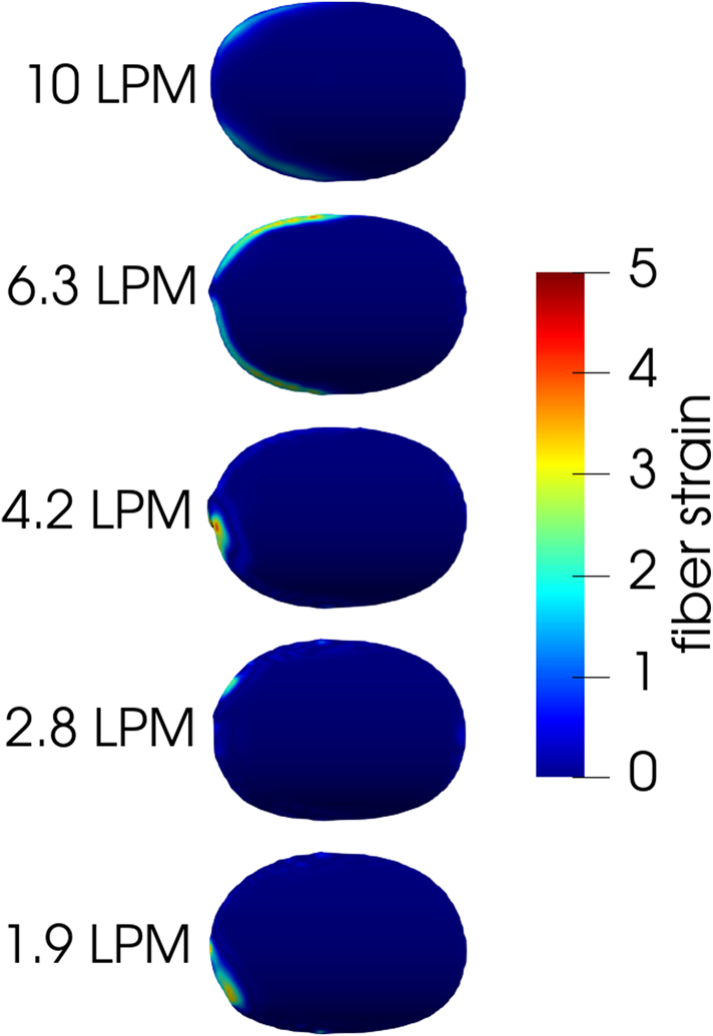
Clots at all flow rates with their lower-bound wall extensibility parameter. For clots that have failed to embolize, fiber strain is still concentrated at the leading edge.

In all cases, simulated clots embolized by detachment of the leading edge. The early embolization at the upper bound value of ${\varepsilon }_{w}=5$ for 4.2 LPM is depicted in Fig. 9. In this figure, the clot is observed from below at several time instances, and is colored by its local fiber strain ($tr\left(\tau \right)\lambda /{\eta }_{p}$). As the clot detaches at the leading edge, a front of high fiber strain (i.e. greater than 10) follows the detachment. Little fiber strain is seen at the trailing edge. Figure 10 depicts clots at all five flow rates colored by their local fiber strain, each at least 10 characteristic time scales after the start of the embolization simulation with their respective lower-bound threshold wall extensibility parameters (i.e. ${\varepsilon }_{w}$ values of 12, 7, 3, 1.7 and 0.3 for flow rates of 1.9, 2.8, 4.2, 6.9 and 10.0 LPM). Here, fiber strain is constrained to the leading edge, and only low values (less than 5) are observed.

#### Grid sensitivity analysis

Consistent with the lower-resolution simulations, the clot in the higher-resolution simulations embolized at a wall extensibility parameter of 15, and failed to embolize at a wall extensibility parameter of 12. The drag on the clot was found to be 2.84 mN, 4.4% higher than for the lower-resolution simulations. This difference is acceptable as sufficiently small as it is under 5%.

## Discussion

**Figure 11:**
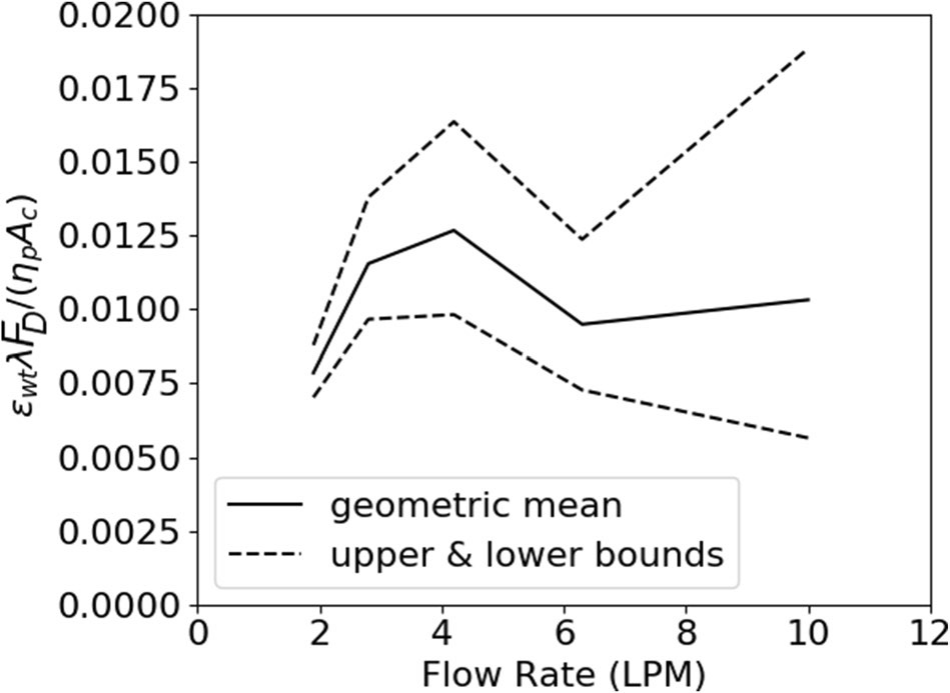
The approximate scaling of the stress breakage argument remains at a constant order of magnitude across flow rates.

The geometric mean threshold wall extensibility parameter decreases logarithmically as a function of flow rate. This is likely due to the form of the breakage function prescribed in Eq. (2), and it may be reasonable for the argument of Eq. (2) to be of the same order of magnitude at the point of embolization across flow rates. If the trace of the stress tensor present in Eq. (2) scales approximately as ${F}_{D}/{A}_{c}$, where ${A}_{c}$ is the area of contact between the clot and the wall, then it may be expected that $\frac{{\varepsilon }_{wt}\lambda {F}_{D}}{{\eta }_{p}{A}_{c}}$ should be approximately constant across flow rate. This quantity is plotted against flow rate in Fig. 11; although the bounds on this quantity are wide, it remains at an order of magnitude of around 0.01 across flow rate. A tighter bounding of the threshold wall extensibility parameter may show a Reynolds number effect, though no such inference can be made with current numerical data.

The experimental flow rates required to embolize the clots in the plain-tube experiments are interpolated onto the simulation data to obtain embolization drag forces and geometric mean threshold wall extensibility parameter data for each experimental embolization. Using this approach gives a mean wall extensibility parameter of 2.92 with a standard deviation of 2.09. Interpolated threshold wall extensibility parameters ranged between 1.51 and 11.01. The embolization drag force had a mean value of 2.28 mN and a standard deviation of 0.93 mN. The embolization drag force ranged between 0.42 mN and 4.89 mN.

Despite the variability in embolization behavior observed in vitro, the in silico clots had very consistent embolization behavior. This is likely due to the non-uniformity in the experimental clots, where lengths ranged between 11.7 and 18.4 mm, in addition to the natural variability in the bovine blood used. In contrast, the clot region was kept identical in all simulations and is purely homogeneous, with no spatial variability in constitutive parameters. Introducing variability into the shape of the in silico clots may lead to different embolization behavior. An especially notable difference between the in silico and in vitro clots is the tendency for in vitro clots to first detach at the trailing edge, while in silico clots first detached at the leading edge, as shown in Supplemental Materials 2. As evidenced by the fiber strains reported in Figs. 9 and 10, there is simply no significant strain concentration at the trailing edge of the in silico clots. The flow at the trailing edge of the clot is possibly not well enough resolved in the simulations, which might explain the low strain in that region. Validation of the fluid flow would help to clarify this discrepancy. An additional possibility is that this discrepancy comes from the assumption in the simulations that the adhesion is uniform throughout the clot. In contrast, it is possible that the in vitro clots had spatially varying adhesion characteristics, both from natural variability in in vitro experiments, and due to potential drying of the clot at the clot-polycarbonate-air interface, forming a ring of dried clot around the perimeter. Finally, it is possible that embolization dynamics were impacted by the movement of the tube during experiments. Although an ideal controlled experiment would have minimal movement of the tube, this complication does highlight the possibility of vessel dynamics on embolization.

The “flag-waving” behavior observed experimentally is an especially interesting dynamic, potentially providing a good validation metric for future refinements in embolization modeling. The lifting of the trailing edge of the clot suggests that there may be chaotic, recirculating flows that play a role in clot detachment.

### Limitations

The disagreement on leading-edge vs trailing-edge separation suggests that the mechanics of embolization are not being fully captured. One possible explanation for this discrepancy is that there is insufficient spatial resolution in the in silico embolizations. The trailing-edge separation and flag-waving behavior suggest that there may be a recirculating eddy downstream of the clot. This was not observed in the simulations, potentially due to the smoothing of the interface between the Newtonian and viscoelastic phases that is necessary to avoid numerical instability. Another potential complication is the pulsatile nature of the peristaltic pump used to drive flow in the experiments leading to the possibility that the slight cyclic nature of the loading on the in vitro clots had some impact on the embolization dynamics.

Numerical issues were a particular challenge in developing the embolization model and precluded some attempts at a more careful analysis. For instance, the requirement for low aspect ratio grid elements combined with the high computational cost of the simulations required near-wall grid elements that only coarsely resolved the viscous boundary layer. Attempts at using local or automated grid refinement similarly met with numerical issues, in particular when the clot interface approached the grid refinement interface. Finally, maintaining the low interface Courant number became costly during the process of embolization, so that the large majority of simulated clots were not allowed to fully embolize, restricting the ability to more fully analyze the mechanics of embolization. Future development of a more numerically stable solution algorithm would be desirable.

In general, it should be expected that adhesion characteristics of blood clots will change depending on the material they are adhered to. The adhesion characteristics of clots to polycarbonate does not necessarily give much information on adhesion characteristics of commonly used biomaterials though this still provides a good foundation to study other biomaterials.

The use in the experiments of statically formed clots in a plain tube does not correlate to any realistic in vivo situation. The flow conditions in which clots are formed heavily impact their composition, which should be expected in turn to impact their mechanical properties and embolization behavior. Because of the role of platelets in clot growth, the concentration of platelets in a clot formed in flow is typically higher than the ambient concentration in circulating blood^29^. However, the use of reconstituted whole blood in the current experiments guarantees that the platelet concentration in the clots is nearly physiological for circulating blood. Applying the current model to an in vivo situation would require a much better understanding of the role of different flow conditions on clot formation and likely heterogenous mechanical property distribution. The experiments and model described here provide a set of conditions and methods to interrogate in vivo conditions in the future.

## Conclusions

A novel modeling approach is introduced for in silico analysis of clot embolization. This approach includes multiphase modeling of a Newtonian fluid and a viscoelastic blood clot, where the constitutive properties of the viscoelastic blood clot are fitted to an in vitro stress–relaxation curve. Adhesion of the clot to the solid surface was modeled by a locally varying extensibility parameter to force the clot to break more aggressively at the wall than in its volume. By varying the flow rate and wall extensibility parameter, the functional dependence of threshold wall extensibility parameter leading to embolization on flow rate was found.

These simulations were compared to in vitro embolization experiments in a geometrically similar setup. Experimental embolization occurred at a mean flow rate of 5.1 LPM, ranging from 2.3 LPM to 7.5 LPM. By interpolating these results onto CFD outputs, it was found that the drag force required to embolize the clot ranged between 0.42 and 4.89 mN, and the wall extensibility parameter ranged between 1.51 and 11.01, suggesting a wide variability of adhesion characteristics in the in vitro experiments.

In silico embolization was found to occur exclusively by separation of the leading edge from the wall, while in vitro embolization was found to more frequently occur by separation of the trailing edge, suggesting some discrepancy between in silico and in vitro stress distribution and embolization behavior and the need for further model development.

### Supplementary Information

Supplementary Information.

Supplementary Video 1.

Supplementary Video 2.

## Data Availability

Data will be made available by the corresponding author (K.B. Manning) upon reasonable request.
