# Supplementary material for: Clot embolization studies and computational framework for embolization in a canonical tube model
Source: Sci Rep. 2023 Sep 6;13:14682. doi: 10.1038/s41598-023-41825-8 (PMC10482921; doi:10.1038/s41598-023-41825-8)
Supplement: Supplementary file 1 — Supplementary Information. [file 41598_2023_41825_MOESM1_ESM.docx]

Specific Implementation Details

The purpose of this supplementary information is to provide sufficiently detailed information on the implementation described in the manuscript for a skilled OpenFOAM practitioner to implement the described model and is assumed that the reader will have some knowledge of running OpenFOAM simulations and modifying OpenFOAM’s source code. More details on the base implementation of the viscoelastic VOF solver can be found in the rheoTool user guide.

Code Modification

Simulations were run using the rheoTool solver rheoInterFoam, which is a volume-of-fluid solver allowing for multiple fluid phases with different constitutive models. A modified version of the PTT constitutiveEq class was coded and compiled that accepted an additional volScalarField “wallAdjacent” and an additional dimensionedScalar “epsilonW”.

The volScalarField wallAdjacent had values of 0 or 1. A custom utility was written that looped through the patches in the OpenFOAM mesh, determined whether their type was “wall”, and set the value of adjacent cells equal to 1 if they had a face on that patch.

The default PTT exponential breakage function was modified as follows to vary the form of the breakage function near the wall:

case pfExpt :

tauEqn += fvm::Sp( (1./lambda)*Foam::exp((epsilon_*(1-wallAdjacent_)+epsilonW_*wallAdjacent_)*lambda/etaP*tr(tau_)), tau_);

break;

Case Setup

Initial and Boundary Conditions

The following pages contain the files included in the zero time directory defining boundary conditions for each patch in the computational domain. As described in the primary manuscript, there were two non-connected regions in the mesh. Region 1 had an inlet patch called “inlet1”, an outlet patch called “outlet1” and a walls patch called “walls1”. Region 2 had similarly named patches. Outlet patches were of type “patch”. Wall patches were of type “wall”. Inlet patches were of type “mappedPatch” in order to map the velocity field from outlet1 to both inlet1 and inlet2. The dictionary setFieldsDict shows the method of setting alpha.clot to initialize the clot shape.

/*--------------------------------*- C++ -*----------------------------------*\

========= |

\\ / F ield | OpenFOAM: The Open Source CFD Toolbox

\\ / O peration | Website: https://openfoam.org

\\ / A nd | Version: 7

\\/ M anipulation |

\*---------------------------------------------------------------------------*/

FoamFile

{

version 2.0;

format ascii;

class volVectorField;

location "0";

object U;

}

// * * * * * * * * * * * * * * * * * * * * * * * * * * * * * * * * * * * * * //

dimensions [1 -1 0 0 0 0 0];

internalField uniform (0 0 0);

boundaryField

{

inlet1

{

type mapped;

field U;

setAverage 1;

average (0.25 0 0);

interpolationScheme cell;

value unifrom (0 0 0);

}

outlet1

{

type inletOutlet;

inletValue uniform (0 0 0);

value uniform (0 0 0);

}

walls1

{

type fixedValue;

value uniform (0 0 0);

}

inlet2

{

type mapped;

field U;

setAverage 0;

average (0.25 0 0);

interpolationScheme cell;

value uniform (0 0 0);

}

outlet2

{

type inletOutlet;

inletValue uniform (0 0 0);

value uniform (0 0 0);

}

walls2

{

type fixedValue;

value uniform (0 0 0);

}

}

// ************************************************************************* //

/*--------------------------------*- C++ -*----------------------------------*\

========= |

\\ / F ield | OpenFOAM: The Open Source CFD Toolbox

\\ / O peration | Website: https://openfoam.org

\\ / A nd | Version: 7

\\/ M anipulation |

\*---------------------------------------------------------------------------*/

FoamFile

{

version 2.0;

format ascii;

class volScalarField;

location "0";

object p_rgh;

}

// * * * * * * * * * * * * * * * * * * * * * * * * * * * * * * * * * * * * * //

dimensions [1 -1 -2 0 0 0 0];

internalField uniform 0;

boundaryField

{

inlet1

{

type zeroGradient;

}

outlet1

{

type fixedValue;

value uniform 0;

}

walls1

{

type fixedFluxPressure;

gradient uniform 0;

value uniform 0;

}

inlet2

{

type zeroGradient;

}

outlet2

{

type fixedValue;

value uniform 0;

}

walls2

{

type fixedFluxPressure;

gradient uniform 0;

value uniform 0;

}

}

// ************************************************************************* //

/*--------------------------------*- C++ -*----------------------------------*\

========= |

\\ / F ield | OpenFOAM: The Open Source CFD Toolbox

\\ / O peration | Website: https://openfoam.org

\\ / A nd | Version: 7

\\/ M anipulation |

\*---------------------------------------------------------------------------*/

FoamFile

{

version 2.0;

format ascii;

class volScalarField;

location "0";

object alpha.clot;

}

// * * * * * * * * * * * * * * * * * * * * * * * * * * * * * * * * * * * * * //

dimensions [0 0 0 0 0 0 0];

internalField uniform 0;

boundaryField

{

inlet1

{

type fixedValue;

value uniform 0;

}

outlet1

{

type zeroGradient;

}

walls1

{

type zeroGradient;

}

inlet2

{

type fixedValue;

value uniform 0;

}

outlet2

{

type zeroGradient;

}

walls2

{

type zeroGradient;

}

}

// ************************************************************************* //

/*--------------------------------*- C++ -*----------------------------------*\

========= |

\\ / F ield | OpenFOAM: The Open Source CFD Toolbox

\\ / O peration | Website: https://openfoam.org

\\ / A nd | Version: 7

\\/ M anipulation |

\*---------------------------------------------------------------------------*/

FoamFile

{

version 2.0;

format ascii;

class volSymmTensorField;

location "0";

object theta.clot;

}

// * * * * * * * * * * * * * * * * * * * * * * * * * * * * * * * * * * * * * //

dimensions [0 0 0 0 0 0 0];

internalField uniform (0 0 0 0 0 0);

boundaryField

{

inlet1

{

type fixedValue;

value uniform (0 0 0 0 0 0);

}

outlet1

{

type zeroGradient;

}

walls1

{

type linearExtrapolation;

value uniform (0 0 0 0 0 0);

}

inlet2

{

type fixedValue;

value uniform (0 0 0 0 0 0);

}

outlet2

{

type zeroGradient;

}

walls2

{

type linearExtrapolation;

value uniform (0 0 0 0 0 0);

}

}

// ************************************************************************* //

/*--------------------------------*- C++ -*----------------------------------*\

========= |

\\ / F ield | OpenFOAM: The Open Source CFD Toolbox

\\ / O peration | Website: https://openfoam.org

\\ / A nd | Version: 7

\\/ M anipulation |

\*---------------------------------------------------------------------------*/

FoamFile

{

version 2.0;

format ascii;

class volSymmTensorField;

location "0";

object tau.clot;

}

// * * * * * * * * * * * * * * * * * * * * * * * * * * * * * * * * * * * * * //

dimensions [1 -1 -2 0 0 0 0];

internalField uniform (0 0 0 0 0 0);

boundaryField

{

inlet1

{

type fixedValue;

value uniform (0 0 0 0 0 0);

}

outlet1

{

type zeroGradient;

}

walls1

{

type linearExtrapolation;

value uniform (0 0 0 0 0 0);

}

inlet2

{

type fixedValue;

value uniform (0 0 0 0 0 0);

}

outlet2

{

type zeroGradient;

}

walls2

{

type linearExtrapolation;

value uniform (0 0 0 0 0 0);

}

}

// ************************************************************************* //

/*--------------------------------*- C++ -*----------------------------------*\

========= |

| \\ / F ield | OpenFOAM: The Open Source CFD Toolbox |

| \\ / O peration | Version: 7.0 |

| \\ / A nd | Website: https://openfoam.org |

| \\/ M anipulation | |

\*---------------------------------------------------------------------------*/

FoamFile

{

version 2.0;

format ascii;

class dictionary;

object setFieldsDict;

}

// * * * * * * * * * * * * * * * * * * * * * * * * * * * * * * * * * * * * * //

defaultFieldValues

(

volScalarFieldValue alpha.clot 0

);

regions

(

cylinderToCell

{

p1 (0.1524 -10 -.01523);

p2 (0.1524 10 -.01523);

radius 0.01143;

fieldValues

(

volScalarFieldValue alpha.clot 1

);

}

);

// ************************************************************************* //

Constitutive Properties

The following shows the “constitutiveProperties” dictionary standard in rheoTool.

/*--------------------------------*- C++ -*----------------------------------*\

========= |

| \\ / F ield | OpenFOAM: The Open Source CFD Toolbox |

| \\ / O peration | Version: 7.0 |

| \\ / A nd | Website: https://openfoam.org |

| \\/ M anipulation | |

\*---------------------------------------------------------------------------*/

FoamFile

{

version 2.0;

format ascii;

class dictionary;

object constitutiveProperties;

}

// * * * * * * * * * * * * * * * * * * * * * * * * * * * * * * * * * * * * * //

phases (clot pbs);

clot

{

parameters

{

type PTTwLog;

rho rho [ 1 -3 0 0 0 0 0 ] 1060;

etaS etaS [1 -1 -1 0 0 0 0] 0;

etaP etaP [1 -1 -1 0 0 0 0] 21000;

lambda lambda [0 0 1 0 0 0 0] 6;

epsilon epsilon [0 0 0 0 0 0 0] 0.01;

epsilonW epsilonW [0 0 0 0 0 0 0] 12;

zeta zeta [0 0 0 0 0 0 0] 0;

destructionFunctionType exponential;

stabilization coupling;

}

}

pbs

{

parameters

{

type Newtonian;

rho rho [1 -3 0 0 0 0 0] 1000;

eta eta [1 -1 -1 0 0 0 0] 1e-3;

}

}

sigma sigma [ 1 0 -2 0 0 0 0 ] 0.0;

// ************************************************************************* //

Solution Control

The following shows the “controlDict” dictionary standard in OpenFOAM. Following this are the fvSolution and fvSchemes dictionaries.

/*--------------------------------*- C++ -*----------------------------------*\

========= |

| \\ / F ield | OpenFOAM: The Open Source CFD Toolbox |

| \\ / O peration | Version: 7.0 |

| \\ / A nd | Website: https://openfoam.org |

| \\/ M anipulation | |

\*---------------------------------------------------------------------------*/

FoamFile

{

version 2.0;

format ascii;

class dictionary;

object controlDict;

}

// * * * * * * * * * * * * * * * * * * * * * * * * * * * * * * * * * * * * * //

application rheoInterFoam;

startFrom latestTime;

startTime 0;

stopAt endTime;

endTime 15.3;

deltaT 0.00000001;

writeControl adjustableRunTime;

writeInterval 0.1;

purgeWrite 1;

writeFormat ascii;

writePrecision 8;

timeFormat general;

timePrecision 8;

runTimeModifiable yes;

adjustTimeStep yes;

maxCo 0.5;

maxAlphaCo 0.01;

maxDeltaT 0.001;

/*--------------------------------*- C++ -*----------------------------------*\

========= |

| \\ / F ield | OpenFOAM: The Open Source CFD Toolbox |

| \\ / O peration | Version: 7.0 |

| \\ / A nd | Website: https://openfoam.org |

| \\/ M anipulation | |

\*---------------------------------------------------------------------------*/

FoamFile

{

version 2.0;

format ascii;

class dictionary;

object fvSolution;

}

// * * * * * * * * * * * * * * * * * * * * * * * * * * * * * * * * * * * * * //

solvers

{

"alpha.clot.*"

{

nAlphaCorr 3;

nAlphaSubCycles 1;

cAlpha 1;

MULESCorr yes;

nLimiterIter 3;

solver smoothSolver;

smoother symGaussSeidel;

tolerance 1e-10;

relTol 0;

}

pcorrFinal

{

solver GAMG;

tolerance 1e-10;

relTol 0;

smoother DIC;

cacheAgglomeration true;

nCellsInCoarsestLevel 100;

agglomerator faceAreaPair;

mergeLevels 1;

}

p_rghFinal

{

solver GAMG;

tolerance 1e-10;

relTol 0;

smoother DIC;

cacheAgglomeration true;

nCellsInCoarsestLevel 100;

agglomerator faceAreaPair;

mergeLevels 1;

}

"(p_rgh|pcorr)"

{

solver GAMG;

tolerance 1e-10;

relTol 0.01;

smoother DIC;

cacheAgglomeration true;

nCellsInCoarsestLevel 100;

agglomerator faceAreaPair;

mergeLevels 1;

}

"(theta.*|tau.*|U)"

{

solver PBiCG;

preconditioner

{

preconditioner DILU;

}

tolerance 1e-10;

relTol 0;

minIter 0;

maxIter 1000;

}

}

PIMPLE

{

nInIter 1;

SIMPLEC true;

nCorrectors 1;

nNonOrthogonalCorrectors 2;

}

relaxationFactors

{

equations

{

".*" 1;

}

}

// ************************************************************************* //

/*--------------------------------*- C++ -*----------------------------------*\

========= |

| \\ / F ield | OpenFOAM: The Open Source CFD Toolbox |

| \\ / O peration | Version: 7.0 |

| \\ / A nd | Website: https://openfoam.org |

| \\/ M anipulation | |

\*---------------------------------------------------------------------------*/

FoamFile

{

version 2.0;

format ascii;

class dictionary;

object fvSchemes;

}

// * * * * * * * * * * * * * * * * * * * * * * * * * * * * * * * * * * * * * //

ddtSchemes

{

default CrankNicolson 0.9;

}

gradSchemes

{

default Gauss linear;

grad(p) Gauss linear;

grad(U) Gauss linear;

linExtrapGrad Gauss linear;

}

divSchemes

{

default none;

div(Sum(tau)) Gauss linear;

div(grad(U)) Gauss linear;

div(rhoPhi,U) Gauss LUST grad(U);

div(phi,alpha) Gauss vanLeer01;

div(phirb,alpha) Gauss interfaceCompression;

div(eta*alpha*dev2(T(gradU))) Gauss linear;

div(phi,theta.clot) GaussDefCmpw cubista;

div(phi,theta.pbs) GaussDefCmpw cubista;

div(phi,tau.clot) GaussDefCmpw cubista;

div(phi,tau.pbs) GaussDefCmpw cubista;

}

laplacianSchemes

{

default Gauss linear corrected;

}

interpolationSchemes

{

default linear;

}

snGradSchemes

{

default corrected;

}

fluxRequired

{

default no;

p_rgh;

pcorr;

alpha1;

}

// ************************************************************************* //
